# Supplementary material for: WDR75: An essential protein for ribosome assembly undergoing purifying selection
Source: PLoS One. 2025 Feb 11;20(2):e0318395. doi: 10.1371/journal.pone.0318395 (PMC11813130; doi:10.1371/journal.pone.0318395)
Supplement: S1 Table — (DOCX) [file pone.0318395.s001.docx]

**Supporting Information**

**WDR75: An essential protein for ribosome assembly undergoing purifying selection**

**Lauren Lee and Justen B. Whittall**

**Table S1. Homologous WDR75 Coding Sequences used in this Study based on the *Homo sapiens* reference (NM_032168)**

| **Sequence # in alignment** | **Organism (Transcript Variant #)** | **Common name** | **GenBank Nucleotide Accession Number^1^** | **Percent Identity (%)^3^** |
| --- | --- | --- | --- | --- |
| 1 | *Homo sapiens* (1) | human | NM_032168^2^ | 100 |
| 2 | *Homo sapiens* | human | BC040567^4^ | 99.96 |
| 3 | *Pan troglodytes* | chimpanzee | XM_001164827 | 99.48 |
| 4 | *Gorilla gorilla gorilla* | gorilla | XM_004032947 | 99.44 |
| 5 | *Homo sapiens* (2) | human | NM_001303096 | 99.35 |
| 6 | *Pan paniscus* (1) | bonobo | XM_003825265 | 99.40 |
| 7 | *Pan paniscus* (2) | bonobo | XM_055108870 | 98.78 |
| 8 | *Pongo abelii* | Sumatran orangutan | XM_002812669 | 98.84 |
| 9 | *Pongo pygmaeus* | Bornean orangutan | XM_054476911 | 98.84 |
| 10 | *Nomascus leucogenys* | northern white-cheeked gibbon | XM_003253841 | 98.80 |
| 11 | *Hylobates moloch* | silvery gibbon | XM_032755184 | 98.68 |
| 12 | *Symphalangus syndactylus* | siamang | XM_055289978 | 98.64 |
| 13 | *Piliocolobus tephrosceles* | Ugandan red Colobus | XM_023212408 | 98.24 |
| 14 | *Papio anubis* | olive baboon | XM_003907720 | 98.16 |
| 15 | *Rhinopithecus roxellana* | golden snub-nosed monkey | XM_010370284 | 98.12 |
| 16 | *Theropithecus gelada* | gelada | XM_025404824 | 98.08 |
| 17 | *Sapajus apella* | tufted capuchin | XM_032292670 | 96.91 |
| 18 | *Cebus imitator* | Panamanian white-faced capuchin | XM_017500740 | 96.83 |
| 19 | *Saimiri boliviensis boliviensis* | Bolivian squirrel monkey | XM_039469944 | 96.03 |
| 20 | *Callithrix jacchus* | white-tufted-ear marmoset | XM_002749555 | 95.91 |
| 21 | *Propithecus coquereli* | Coquerel's sifaka | XM_012663655 | 92.94 |
| 22 | *Lemur catta* | Ring-tailed lemur | XM_045560137 | 92.90 |
| 23 | *Lynx rufus* | bobcat | XM_047084122 | 92.62 |
| 24 | *Felis Catus* | domestic cat | XM_003990963 | 92.62 |
| 25 | *Acinonyx jubatus* | cheetah | XM_015077655 | 92.58 |
| 26 | *Puma yagouaroundi* | jaguarundi | XM_040475624 | 92.50 |
| 27 | *Panthera onca* | jaguar | XM_060622310 | 92.46 |
| 28 | *Neomonachus schauinslandi* | Hawaiian monk seal | XM_021703388 | 92.42 |
| 29 | *Panthera tigris* | tiger | XM_042994893 | 92.38 |
| 30 | *Eumetopias jubatus* | steller sea lion | XM_028125109 | 92.34 |
| 31 | *Equus quagga* | plains zebra | XM_046659327 | 92.30 |
| 32 | *Callorhinus ursinus* | northern fur seal | XM_025848764 | 92.22 |
| 33 | *Carlito syrichta* | Philippine tarsier | XM_008062665 | 92.18 |
| 34 | *Mirounga leonina* | Southern elephant seal | XM_035007552 | 92.14 |
| 35 | *Mirounga angustirostris* | Northern elephant seal | XM_045868369 | 92.10 |
| 36 | *Pteropus giganteus* | Indian flying fox | XM_039844223 | 91.94 |
| 37 | *Nycticebus coucang* | slow loris | XM_053597378 | 91.94 |
| 38 | *Hyaena hyaena* | striped hyena | XM_039224335 | 91.90 |
| 39 | *Orcinus orca* | killer whale | XM_004276927 | 91.87 |
| 40 | *Delphinus Delphis* | dolphin | XM_060016452 | 91.83 |
| 41 | *Otolemur garnettii* | small-eared galago | XM_003801032 | 91.70 |
| 42 | *Halichoerus grypus* | gray seal | XM_036119966 | 91.43 |
| 43 | *Suricata suricatta* | meerkat | XM_029934265 | 91.22 |
| 44 | *Camelus dromedarius* | Arabian camel | XM_010991855 | 91.16 |
| 45 | *Marmota flaviventris* | yellow- bellied marmot | XM_027925024 | 91.10 |
| 46 | *Urocitellus parryii* | Arctic ground squirrel | XM_026389072 | 91.10 |
| 47 | *Sus scrofa* | pig | XM_003133536 | 91.11 |
| 48 | *Camelus ferus* | Wild Bactrian camel | XM_006189230 | 91.12 |
| 49 | *Ictidomys tridecemlineatus* | thirteen-lined ground squirrel | XM_005324385 | 91.02 |
| 50 | *Manis pentadactyla* | Chinese pangolin | XM_036881688 | 91.02 |
| 51 | *Marmota marmota marmota* | Alpine marmot | XM_015481143 | 90.98 |
| 52 | *Molossus molossus* | Pallas's mastiff bat | XM_036254888 | 91.55 |
| 53 | *Rousettus aegyptiacus* | Egyptian rousette | XM_016148700 | 91.30 |
| 54 | *Rhinolophus ferrumequinum* | greater horseshoe bat | XM_033112205 | 91.30 |
| 55 | *Rhinolophus sinicus* | Chinese rufous horseshoe bat | XM_019740758 | 91.30 |
| 56 | *Miniopterus natalensis* | natal long-fingered bat | XM_016214313 | 91.19 |
| 57 | *Canis lupus familiaris* | dog | XM_545565 | 90.05 |
| 58 | *Vulpes lagopus* | Artic fox | XM_041737665 | 89.97 |
| 59 | *Phyllostomus discolor* | pale spear-nosed bat | XM_028509773 | 90.17 |
| 60 | *Pteronotus parnellii mesoamericanus* | Parnell's mustached bat | XM_054562310 | 89.93 |
| 61 | *Desmodus rotundus* | common vampire bat | XM_024564006 | 89.53 |
| 62 | *Artibeus jamaicensis* | Jamaican fruit-eating bat | XM_037162931 | 89.49 |
| 63 | *Sturnira hondurensis* | Honduran yellow-shouldered bat | XM_037043776 | 89.17 |
| 64 | *Mesocricetus auratus* | golden hamster | XM_005084482 | 84.48 |
| 65 | *Peromyscus maniculatus bairdii* | prairie deer mouse | XM_006974841 | 83.93 |
| 66 | *Onychomys torridus* | southern grasshopper mouse | XM_036173141 | 83.88 |
| 67 | *Microtus oregoni* | creeping vole | XM_041668403 | 79.66 |
| 68 | *Myotis myotis* | greater mouse-eared bat | XM_036319609 | 77.41 |
| 69 | *Myotis daubentonii* | Daubenton's bat | XM_059702534 | 77.33 |
| 70 | *Myotis davidii* | David's mouse-eared bat | XM_006767058 | 75.14 |
| 71 | *Caretta caretta^5^* | loggerhead turtle | XM_048868619 | 71.51 |
| 72 | *Chrysemys picta bellii^5^* | western painted turtle | XM_005308040 | 71.48 |
| 73 | *Alligator mississippiensis^5^* | American alligator | XM_006268745 | 71.44 |
| 74 | *Mauremys reevesii^5^* | Reeve’s Turtle | XM_039495578 | 71.47 |
| 75 | *Chelonia mydas^5^* | green sea turtle | XM_007069897 | 71.43 |

^1^ Sequences associated with an E-value of 0 and were found using a blastn search

^2^ Reference sequence

^3^ Sequences sorted by # of differences to reference sequence.

^4^ From nucleotide collection database

^5^ Outgroup
