# Supplementary material for: WDR75: An essential protein for ribosome assembly undergoing purifying selection
Source: PLoS One. 2025 Feb 11;20(2):e0318395. doi: 10.1371/journal.pone.0318395 (PMC11813130; doi:10.1371/journal.pone.0318395)
Supplement: S2 Table — (DOCX) [file pone.0318395.s002.docx]

**Supporting Information**

**WDR75: An essential protein for ribosome assembly undergoing purifying selection**

**Lauren Lee and Justen B. Whittall**

**Table S2. Mammalian WDR75 nucleotide and protein alignment files.**

| Alignment File Name | Description |
| --- | --- |
| Ingroup alignment.fasta | Nucleotide alignment, ingroup only with stop codon |
| Ingroup translation alignment.fasta | Protein alignment, ingroup only with stop codon |
| Ingroup for dNdS.fasta | Nucleotide alignment, ingroup only without stop codon |
| Ingroup and outgroup.fasta | Nucleotide alignment, ingroup and reptile outgroup for phylogenetic analysis |
| Ingroup and outgroup myotis deletion.fasta | Nucleotide alignment, ingroup and reptile outgroup with the uniquely inserted three codons in *Myotis* removed (at reference sequence DNA site 2419bp). |
