# Supplementary material for: WDR75: An essential protein for ribosome assembly undergoing purifying selection
Source: PLoS One. 2025 Feb 11;20(2):e0318395. doi: 10.1371/journal.pone.0318395 (PMC11813130; doi:10.1371/journal.pone.0318395)
Supplement: S3 Table — (DOCX) [file pone.0318395.s003.docx]

**Supporting Information**

**WDR75: An essential protein for ribosome assembly undergoing purifying selection**

**Lauren Lee and Justen B. Whittall**

**Table S3. Amino acid sites exhibiting significant purifying selection using Datamonkey’s SLAC online server for the entire WDR75 coding sequence across mammals.**

| **Amino Acid Site** | **dN-dS** | **P [dN/dS < 1]** |  | **Amino Acid Site** | **dN-dS** | **P [dN/dS < 1]** |  | **Amino Acid Site** | **dN-dS** | **P [dN/dS < 1]** |
| --- | --- | --- | --- | --- | --- | --- | --- | --- | --- | --- |
| 5 | -3.135 | 0.019 |  | 280 | -3.419 | 0.019 |  | 537 | -2.329 | 0.015 |
| 12 | -2.896 | 0.004 |  | 284 | -3.365 | 0.012 |  | 547 | -2.041 | 0.019 |
| 13 | -3.516 | 0.008 |  | 287 | -3.204 | 0.003 |  | 550 | -2.286 | 0.013 |
| 14 | -4.804 | 0.000 |  | 288 | -2.621 | 0.006 |  | 553 | -4.572 | 0.000 |
| 15 | -2.317 | 0.012 |  | 289 | -4.001 | 0.000 |  | 554 | -2.365 | 0.017 |
| 16 | -1.738 | 0.037 |  | 290 | -2.283 | 0.012 |  | 555 | -5.144 | 0.000 |
| 19 | -3.726 | 0.006 |  | 291 | -4.221 | 0.001 |  | 557 | -2.364 | 0.019 |
| 20 | -4.121 | 0.001 |  | 292 | -3.429 | 0.002 |  | 567 | -3.649 | 0.001 |
| 21 | -7.071 | 0.000 |  | 295 | -3.000 | 0.010 |  | 569 | -2.333 | 0.017 |
| 22 | -3.031 | 0.003 |  | 296 | -2.830 | 0.017 |  | 571 | -3.157 | 0.003 |
| 23 | -1.918 | 0.030 |  | 297 | -6.355 | 0.000 |  | 573 | -1.554 | 0.050 |
| 24 | -2.896 | 0.020 |  | 299 | -4.750 | 0.003 |  | 574 | -3.898 | 0.001 |
| 26 | -2.480 | 0.013 |  | 300 | -2.286 | 0.012 |  | 578 | -4.158 | 0.002 |
| 27 | -3.475 | 0.001 |  | 301 | -6.287 | 0.000 |  | 580 | -2.080 | 0.048 |
| 28 | -4.054 | 0.000 |  | 302 | -4.001 | 0.000 |  | 582 | -3.234 | 0.004 |
| 30 | -4.634 | 0.000 |  | 303 | -5.144 | 0.000 |  | 584 | -3.214 | 0.047 |
| 36 | -2.879 | 0.004 |  | 304 | -2.858 | 0.004 |  | 587 | -3.429 | 0.001 |
| 37 | -3.455 | 0.001 |  | 305 | -2.360 | 0.014 |  | 589 | -2.406 | 0.015 |
| 38 | -1.727 | 0.041 |  | 306 | -2.949 | 0.030 |  | 590 | -2.000 | 0.045 |
| 40 | -2.350 | 0.015 |  | 308 | -3.117 | 0.004 |  | 591 | -2.363 | 0.014 |
| 43 | -2.303 | 0.012 |  | 309 | -2.858 | 0.004 |  | 592 | -5.144 | 0.001 |
| 44 | -5.728 | 0.001 |  | 310 | -2.858 | 0.004 |  | 594 | -2.286 | 0.019 |
| 48 | -1.727 | 0.037 |  | 312 | -3.429 | 0.001 |  | 596 | -5.733 | 0.000 |
| 50 | -3.084 | 0.004 |  | 314 | -6.376 | 0.000 |  | 598 | -1.715 | 0.037 |
| 52 | -4.031 | 0.000 |  | 316 | -6.231 | 0.001 |  | 599 | -4.572 | 0.000 |
| 53 | -2.470 | 0.013 |  | 317 | -4.010 | 0.017 |  | 601 | -1.715 | 0.037 |
| 54 | -3.778 | 0.028 |  | 318 | -3.933 | 0.013 |  | 603 | -2.571 | 0.018 |
| 55 | -5.291 | 0.000 |  | 322 | -4.056 | 0.001 |  | 606 | -1.715 | 0.037 |
| 56 | -3.045 | 0.044 |  | 323 | -2.697 | 0.005 |  | 608 | -4.060 | 0.001 |
| 57 | -2.879 | 0.004 |  | 326 | -7.789 | 0.000 |  | 611 | -3.200 | 0.006 |
| 58 | -3.288 | 0.003 |  | 328 | -1.715 | 0.037 |  | 612 | -2.329 | 0.015 |
| 63 | -4.313 | 0.001 |  | 329 | -2.575 | 0.008 |  | 613 | -2.150 | 0.049 |
| 66 | -5.770 | 0.002 |  | 333 | -3.429 | 0.001 |  | 614 | -1.715 | 0.037 |
| 71 | -3.801 | 0.003 |  | 335 | -3.198 | 0.003 |  | 615 | -2.846 | 0.015 |
| 75 | -4.047 | 0.001 |  | 340 | -4.001 | 0.000 |  | 617 | -2.286 | 0.012 |
| 77 | -2.286 | 0.012 |  | 345 | -2.344 | 0.014 |  | 618 | -2.366 | 0.011 |
| 83 | -3.429 | 0.001 |  | 351 | -2.648 | 0.012 |  | 619 | -1.715 | 0.037 |
| 89 | -2.322 | 0.020 |  | 353 | -2.238 | 0.024 |  | 623 | -2.831 | 0.035 |
| 94 | -1.883 | 0.044 |  | 356 | -3.849 | 0.001 |  | 634 | -1.715 | 0.041 |
| 104 | -1.694 | 0.038 |  | 358 | -2.286 | 0.012 |  | 635 | -1.715 | 0.037 |
| 108 | -2.604 | 0.036 |  | 359 | -3.250 | 0.003 |  | 637 | -3.429 | 0.001 |
| 114 | -2.690 | 0.020 |  | 365 | -2.748 | 0.005 |  | 638 | -3.135 | 0.007 |
| 115 | -3.615 | 0.004 |  | 366 | -4.376 | 0.002 |  | 641 | -2.003 | 0.045 |
| 133 | -2.286 | 0.017 |  | 369 | -2.957 | 0.005 |  | 643 | -3.211 | 0.026 |
| 139 | -5.383 | 0.000 |  | 370 | -2.918 | 0.013 |  | 644 | -2.728 | 0.033 |
| 142 | -5.093 | 0.001 |  | 371 | -1.958 | 0.042 |  | 647 | -2.858 | 0.006 |
| 145 | -3.206 | 0.004 |  | 372 | -3.241 | 0.005 |  | 651 | -2.187 | 0.039 |
| 146 | -3.143 | 0.007 |  | 373 | -2.331 | 0.015 |  | 654 | -3.259 | 0.003 |
| 155 | -4.526 | 0.002 |  | 384 | -3.112 | 0.004 |  | 655 | -3.779 | 0.001 |
| 159 | -2.286 | 0.018 |  | 386 | -1.715 | 0.037 |  | 656 | -3.429 | 0.001 |
| 160 | -1.715 | 0.037 |  | 389 | -2.377 | 0.031 |  | 662 | -1.715 | 0.037 |
| 165 | -3.156 | 0.003 |  | 390 | -2.692 | 0.027 |  | 663 | -3.843 | 0.012 |
| 167 | -2.877 | 0.046 |  | 391 | -5.287 | 0.000 |  | 668 | -1.884 | 0.044 |
| 170 | -3.900 | 0.011 |  | 392 | -2.390 | 0.017 |  | 671 | -2.353 | 0.014 |
| 171 | -3.886 | 0.001 |  | 396 | -5.144 | 0.000 |  | 675 | -1.715 | 0.037 |
| 172 | -3.792 | 0.002 |  | 397 | -3.113 | 0.004 |  | 677 | -3.729 | 0.001 |
| 174 | -1.715 | 0.037 |  | 404 | -2.052 | 0.019 |  | 681 | -1.715 | 0.037 |
| 175 | -2.008 | 0.045 |  | 406 | -5.715 | 0.000 |  | 684 | -2.935 | 0.015 |
| 176 | -1.911 | 0.027 |  | 407 | -2.858 | 0.004 |  | 687 | -2.055 | 0.031 |
| 178 | -2.080 | 0.048 |  | 410 | -2.645 | 0.006 |  | 693 | -7.693 | 0.000 |
| 181 | -2.858 | 0.004 |  | 416 | -2.175 | 0.020 |  | 695 | -1.715 | 0.037 |
| 182 | -2.858 | 0.004 |  | 417 | -3.374 | 0.005 |  | 696 | -2.858 | 0.004 |
| 190 | -2.286 | 0.012 |  | 420 | -4.000 | 0.003 |  | 700 | -4.791 | 0.010 |
| 192 | -2.542 | 0.032 |  | 426 | -4.613 | 0.005 |  | 701 | -2.548 | 0.014 |
| 193 | -2.319 | 0.015 |  | 428 | -2.183 | 0.020 |  | 702 | -2.286 | 0.014 |
| 194 | -2.005 | 0.045 |  | 430 | -1.715 | 0.037 |  | 703 | -3.395 | 0.007 |
| 195 | -2.325 | 0.022 |  | 435 | -2.286 | 0.012 |  | 705 | -3.277 | 0.006 |
| 202 | -2.361 | 0.016 |  | 436 | -2.398 | 0.014 |  | 720 | -4.745 | 0.000 |
| 203 | -2.605 | 0.035 |  | 437 | -2.286 | 0.012 |  | 723 | -4.708 | 0.001 |
| 204 | -2.571 | 0.043 |  | 442 | -2.286 | 0.012 |  | 724 | -3.291 | 0.003 |
| 208 | -4.727 | 0.000 |  | 443 | -2.335 | 0.015 |  | 726 | -2.334 | 0.022 |
| 209 | -2.858 | 0.004 |  | 448 | -2.858 | 0.004 |  | 727 | -2.858 | 0.004 |
| 212 | -3.143 | 0.007 |  | 454 | -2.329 | 0.015 |  | 730 | -6.613 | 0.001 |
| 213 | -2.346 | 0.017 |  | 459 | -2.162 | 0.048 |  | 731 | -2.572 | 0.018 |
| 214 | -2.450 | 0.013 |  | 462 | -2.882 | 0.040 |  | 732 | -3.429 | 0.001 |
| 215 | -4.001 | 0.000 |  | 465 | -1.715 | 0.037 |  | 736 | -4.673 | 0.000 |
| 218 | -4.023 | 0.001 |  | 467 | -2.376 | 0.014 |  | 738 | -2.183 | 0.043 |
| 220 | -5.489 | 0.000 |  | 469 | -3.148 | 0.003 |  | 739 | -2.286 | 0.012 |
| 222 | -2.286 | 0.045 |  | 474 | -4.572 | 0.000 |  | 740 | -2.286 | 0.012 |
| 223 | -1.715 | 0.037 |  | 479 | -3.107 | 0.004 |  | 741 | -1.715 | 0.037 |
| 228 | -3.876 | 0.001 |  | 480 | -2.373 | 0.014 |  | 742 | -5.520 | 0.000 |
| 229 | -2.570 | 0.008 |  | 482 | -2.330 | 0.015 |  | 743 | -4.572 | 0.000 |
| 230 | -3.408 | 0.001 |  | 483 | -4.952 | 0.002 |  | 744 | -2.052 | 0.031 |
| 231 | -1.715 | 0.037 |  | 484 | -4.060 | 0.001 |  | 745 | -5.144 | 0.000 |
| 233 | -4.177 | 0.000 |  | 487 | -2.286 | 0.012 |  | 747 | -2.286 | 0.012 |
| 241 | -5.693 | 0.000 |  | 491 | -1.715 | 0.037 |  | 751 | -2.453 | 0.015 |
| 242 | -2.586 | 0.017 |  | 493 | -3.260 | 0.003 |  | 755 | -2.928 | 0.046 |
| 243 | -4.897 | 0.000 |  | 494 | -3.101 | 0.004 |  | 756 | -3.480 | 0.029 |
| 244 | -4.572 | 0.000 |  | 495 | -2.286 | 0.012 |  | 757 | -5.715 | 0.000 |
| 246 | -1.867 | 0.046 |  | 496 | -2.286 | 0.012 |  | 758 | -4.019 | 0.001 |
| 251 | -2.329 | 0.015 |  | 498 | -7.014 | 0.000 |  | 759 | -2.033 | 0.032 |
| 253 | -1.715 | 0.037 |  | 499 | -2.451 | 0.013 |  | 760 | -2.827 | 0.007 |
| 257 | -1.715 | 0.037 |  | 500 | -3.846 | 0.001 |  | 761 | -3.425 | 0.009 |
| 261 | -2.567 | 0.019 |  | 501 | -2.405 | 0.018 |  | 764 | -3.715 | 0.009 |
| 264 | -5.471 | 0.000 |  | 503 | -1.715 | 0.037 |  | 773 | -2.072 | 0.049 |
| 265 | -2.044 | 0.031 |  | 504 | -2.286 | 0.012 |  | 776 | -35.884 | 0.005 |
| 267 | -4.673 | 0.000 |  | 506 | -2.364 | 0.017 |  | 778 | -3.761 | 0.004 |
| 268 | -5.715 | 0.000 |  | 509 | -3.429 | 0.001 |  | 785 | -2.858 | 0.007 |
| 269 | -5.144 | 0.000 |  | 511 | -3.892 | 0.001 |  | 790 | -2.845 | 0.015 |
| 270 | -6.951 | 0.000 |  | 516 | -2.858 | 0.004 |  | 806 | -3.239 | 0.003 |
| 271 | -7.489 | 0.000 |  | 519 | -2.333 | 0.015 |  | 819 | -2.331 | 0.016 |
| 273 | -4.001 | 0.000 |  | 520 | -2.211 | 0.019 |  | 820 | -3.610 | 0.005 |
| 274 | -5.684 | 0.000 |  | 521 | -2.407 | 0.015 |  | 826 | -2.957 | 0.004 |
| 275 | -7.430 | 0.000 |  | 522 | -3.663 | 0.031 |  | 828 | -4.441 | 0.002 |
| 276 | -2.179 | 0.020 |  | 528 | -2.535 | 0.026 |  | 830 | -5.707 | 0.000 |
| 278 | -7.412 | 0.000 |  | 532 | -2.404 | 0.015 |  |  |  |  |
| 279 | -3.927 | 0.009 |  | 536 | -3.429 | 0.001 |  |  |  |  |
