# Supplementary material for: WDR75: An essential protein for ribosome assembly undergoing purifying selection
Source: PLoS One. 2025 Feb 11;20(2):e0318395. doi: 10.1371/journal.pone.0318395 (PMC11813130; doi:10.1371/journal.pone.0318395)
Supplement: S2 Fig — Green branches identify the reptile outgroups rooted with Alligator mississippiensis. Chiroptera (red), Rodentia (blue), and primates (purple) are colored branches and tip labels. The remaining mammals, mostly Carnivora and Artiodactyla, are shown in black. (DOCX) [file pone.0318395.s005.docx]

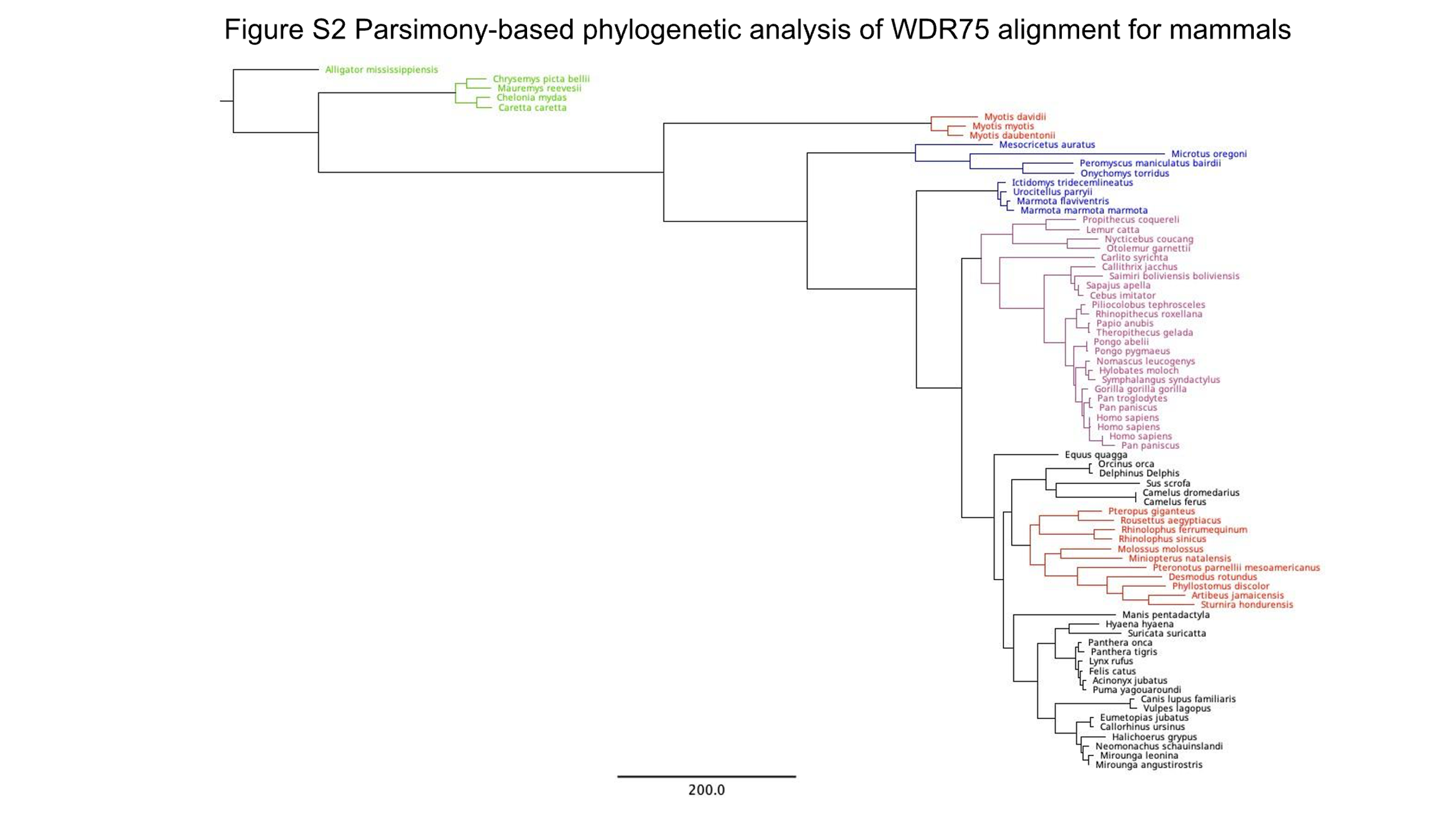


**Fig. S2 Parsimony-based phylogenetic analysis of WDR75 alignment for mammals.** Green branches identify the reptile outgroups rooted with *Alligator mississippiensis*. Chiroptera (red), Rodentia (blue), and primates (purple) are colored branches and tip labels. The remaining mammals, mostly Carnivora and Artiodactyla, are shown in black.
