# Supplementary material for: WDR75: An essential protein for ribosome assembly undergoing purifying selection
Source: PLoS One. 2025 Feb 11;20(2):e0318395. doi: 10.1371/journal.pone.0318395 (PMC11813130; doi:10.1371/journal.pone.0318395)
Supplement: S3 Fig — Panel A shows the predicted human WDR75 3D structures and panel B shows the predicted bonobo WDR75 3D structures. Blue structures are the complete reference sequences while yellow structures indicate the transcript variants that are shortened on the N-terminus. (DOCX) [file pone.0318395.s006.docx]

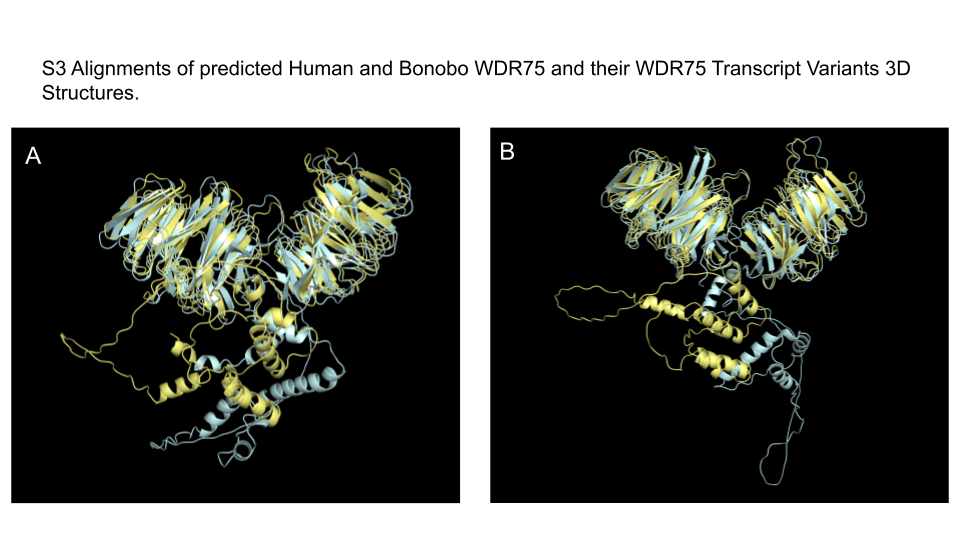


**Fig. S3. Alpha-fold predicted 3D structures of human and bonobo WDR75 aligned with their transcript variants.** Panel A shows the predicted human WDR75 3D structures and panel B shows the predicted bonobo WDR75 3D structures. Blue structures are the complete reference sequences while yellow structures indicate the transcript variants that are shortened on the N-terminus.
