# Supplementary material for: WDR75: An essential protein for ribosome assembly undergoing purifying selection
Source: PLoS One. 2025 Feb 11;20(2):e0318395. doi: 10.1371/journal.pone.0318395 (PMC11813130; doi:10.1371/journal.pone.0318395)
Supplement: S4 Fig — Green branches identify the reptile outgroups rooted with Alligator mississippiensis. Chiroptera (red), Rodentia (blue), and primates (purple) are colored branches and tip labels. The remaining mammals, mostly Carnivora and Artiodactyla, are shown in black. The long branches are still present even though the inserted codons in Myotis were removed. (DOCX) [file pone.0318395.s007.docx]

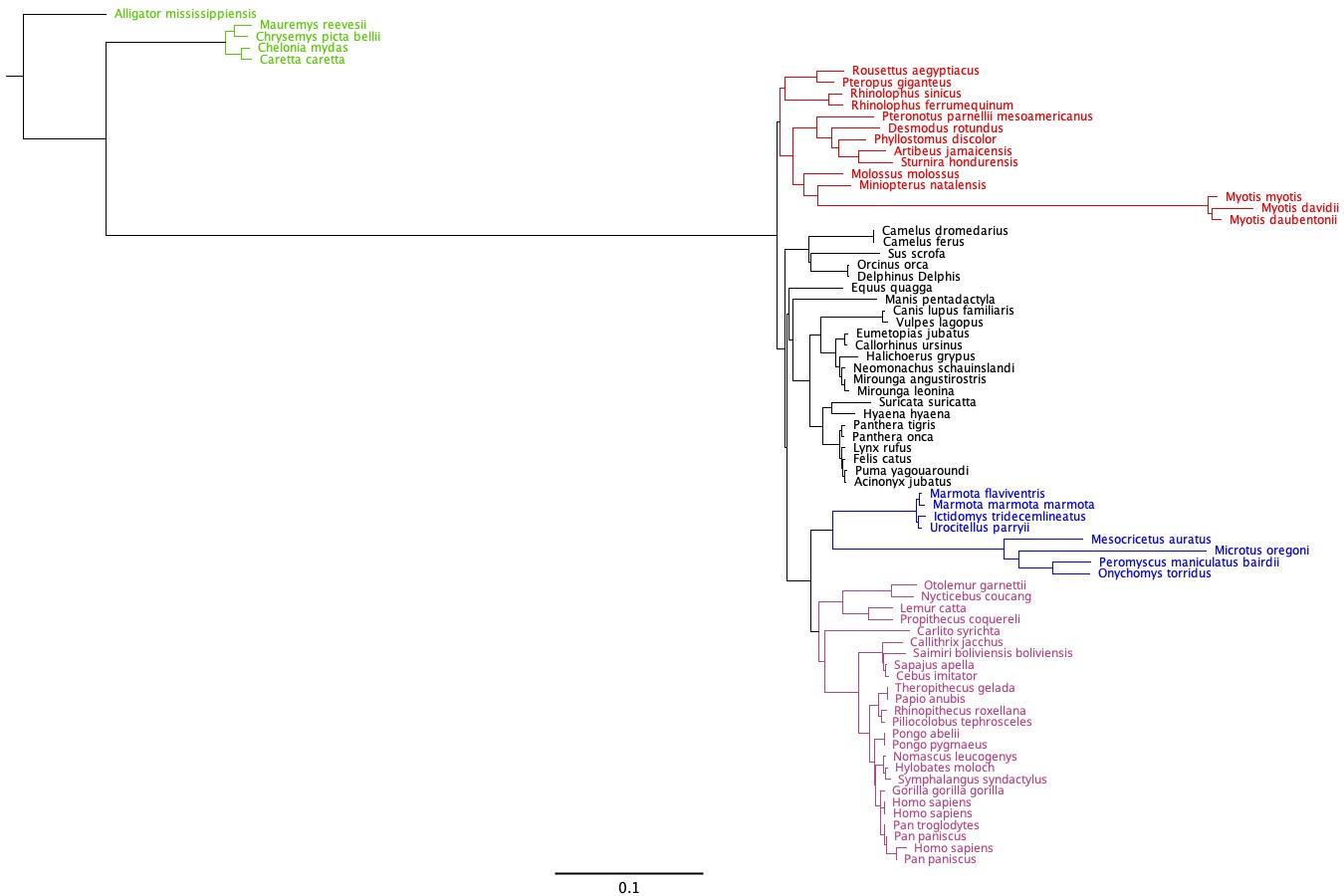


**Fig. S4 Maximum likelihood phylogenetic analysis of WDR75 alignment for mammals with the additional three codons in *Myotis* removed (at reference sequence DNA site 2419).** Green branches identify the reptile outgroups rooted with *Alligator mississippiensis*. Chiroptera (red), Rodentia (blue), and primates (purple) are colored branches and tip labels. The remaining mammals, mostly Carnivora and Artiodactyla, are shown in black. The long branches are still present even though the inserted codons in *Myotis* were removed.
