# Supplementary material for: WDR75: An essential protein for ribosome assembly undergoing purifying selection
Source: PLoS One. 2025 Feb 11;20(2):e0318395. doi: 10.1371/journal.pone.0318395 (PMC11813130; doi:10.1371/journal.pone.0318395)
Supplement: S5 Fig — Homo sapiens RefSeq (NM_032168) is the reference in yellow at the top. Dots indicate identical amino acids to the reference. Box A shows amino acid sites 237–246 (bp 709–728), B shows amino acid site 288 (bp 862–864), and C contains amino acids 321 and 322 (bp 961–966) (see Fig 2 for alignment wide location of A, B, C). Note: there is a break in the alignment between regions A and B+C. (DOCX) [file pone.0318395.s008.docx]

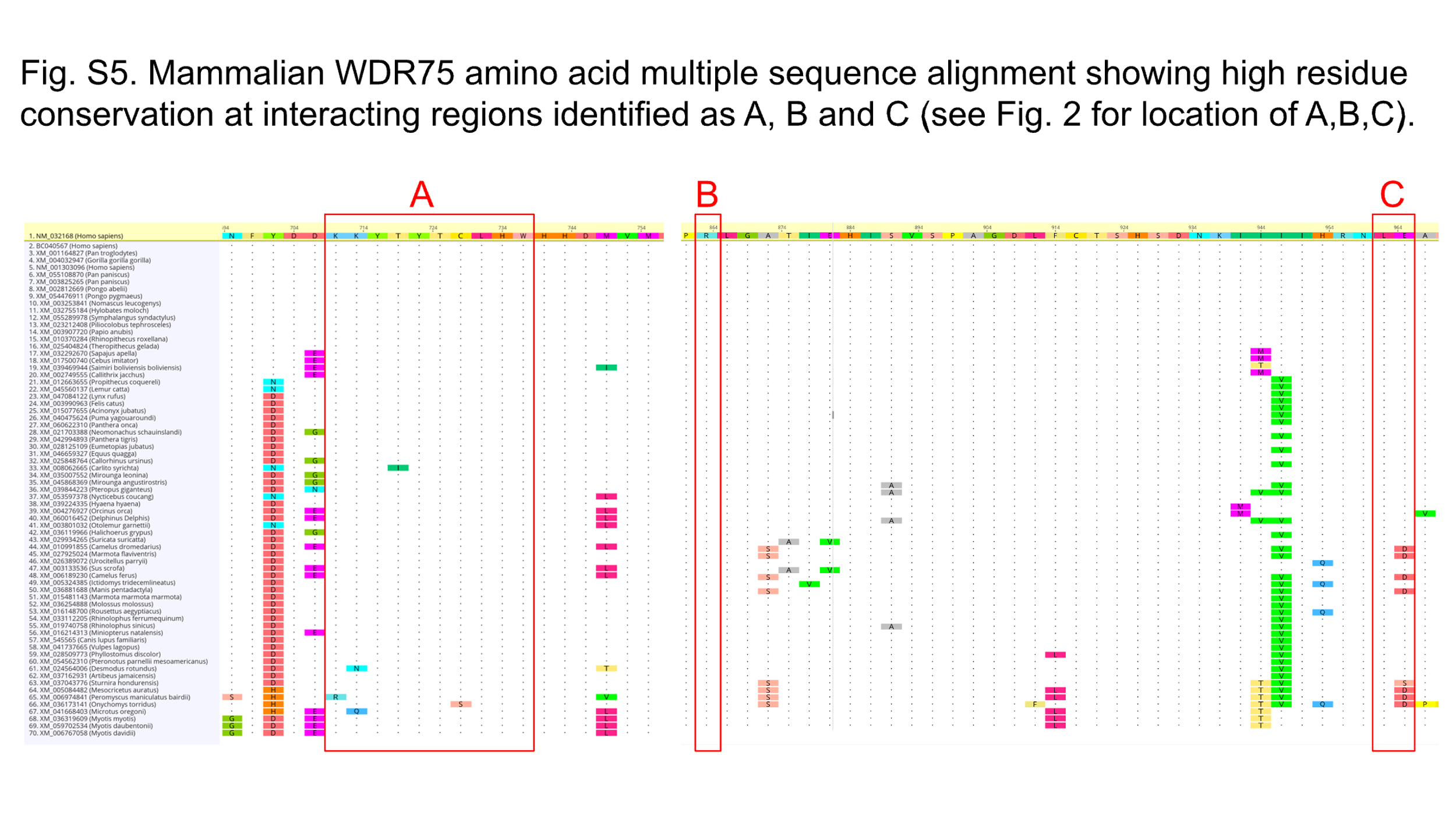


**Fig. S5. Mammalian WDR75 amino acid multiple sequence alignment showing high residue conservation at interacting sites.** *Homo sapiens* RefSeq (NM_032168) is the reference in yellow at the top. Dots indicate identical amino acids to the reference. Box A shows amino acid sites 237-246 (bp 709-728), B shows amino acid site 288 (bp 862-864), and C contains amino acids 321 and 322 (bp 961-966) (see Fig. 2 for alignment wide location of A,B,C). Note: there is a break in the alignment between regions A and B+C.
