# Supplementary material for: WDR75: An essential protein for ribosome assembly undergoing purifying selection
Source: PLoS One. 2025 Feb 11;20(2):e0318395. doi: 10.1371/journal.pone.0318395 (PMC11813130; doi:10.1371/journal.pone.0318395)
Supplement: Attachment — Submitted filename: Response to Reviewers letter.resubmitted.docx [file pone.0318395.s010.docx]

Sept 28, 2024

Dear PLOS ONE Editor,

Thank you for sharing the two reviews of our manuscript (PONE-D-24-35385) entitled, “WDR75: An essential protein for ribosome assembly undergoing purifying selection.” We are grateful for the critical evaluations and especially the 23 points raised by Reviewer #1, most of which we integrated into this revised version. Below you will find the editor’s comments followed by both reviewers’ comments. Our responses to their suggestions are in **bold** font below each comment.

Thank you for reconsidering our manuscript as a Research Paper at *PLOS ONE*. It currently measures ~6,600 words from the beginning of the Abstract to the end of the References (including two tables and seven figure captions). We also have Supplemental Data including three supplemental tables and three supplemental figures, three of which are new or substantially revised (Table S2, Fig. S3, Fig. S4).

Journal Requirements

1. When submitting your revision, we need you to address these additional requirements. Please ensure that your manuscript meets PLOS ONE's style requirements, including those for file naming.

**Done**

2. We note that you have included the phrase “data not shown” in your manuscript. Unfortunately, this does not meet our data sharing requirements.

**We have removed “data not shown” and replaced it with a reference to a new Supplemental Fig. S4 which shows the effect of removing the three codons that were unique to *Myotis* and still exhibits an exceptionally long branch.**

Reviewer #1 Comments:

1-The authors consistently call the SSU processome a 71-protein multimer. It is not clear till later in the paper as to why they use the number 71 - which could be the number of proteins in the human SSU processome cryoEM paper. In yeast, there might be as many as 75 proteins in the SSU processome, though some of these are yeast-specific proteins. Regardless, other than a mention that there are 71 to 75 proteins in the SSU processome, the complex should be called by its proper name, which is the SSU processome not the 71-protein multimer.

**We have limited our reference to the number of proteins in the SSU processome to avoid confusion. When stated as 71-multimer it is always qualified as “in humans”. In the Introduction, we have removed several references to the 71-protein multimer and refer to it simply as the SSU processome as suggested.**

**We also added the number of proteins in the yeast SSU processome to the Introduction by writing, “Within this intricate landscape of ribosomal biology, the small subunit (SSU) processome stands out as a dynamic ensemble of ribonucleoprotein complexes, composed of 71 proteins in humans and 75 proteins in yeast.”**

2-In the abstract, line 21, it is incorrect to call WDR75 a ribosomal protein. It is not a ribosomal protein, but rather an SSU processome protein.

**Good catch. Our mistake. Fixed.**

3-Many of the references in the ms need to be updated with more recent references.

**We appreciate the suggestion and have added two references from within the last six years.**

- **Vincent et al. (2018)**
- **Mirdita et al. (2022)**
- **Gallagher (2019)**

4-Lines 49/50-While Diamond-Blackfan anemia is likely the most common ribosomopathy, North American Indian Childhood cirrhosis is not common and is in fact quite rare. For that reason, I would remove it from that sentence. The authors might choose to keep it though because their protein of interest, WDR75/Utp17 is a member of the UTP-A/t-Utp subcomplex (a fundamental concept that should be included in this paper but is not) and North American Indian Childhood cirrhosis is a ribosomopathy affecting a different member of the Utp-A/t-Utp subcomplex. This disorder should aslo be referred to by its newer name, Progressive cholestasis of northwestern Quebec (PCNQ). It should also be noted that while Diamond-Blackfan anemia is in a ribosomal protein, NAIC/PCNQ is in an SSU processome protein. Lastly, human diseases should not be called mutations but rather variants.

**Thank you for clarifying these details regarding ribosomopathies. As suggested, we have included a reference to WDR75 being part of the UTP-A/t-Utp subcomplex in the Introduction “WDR75 is a key component of both the transcription-Utp complex (t-Utp) and the UtpA complex. The t-Utp complex is crucial for the transcription and early processing of pre-rRNA, while the UtpA complex represents proteins that are part of the mature SSU processome assembly (Gallagher, 2019).”**

**We have replaced NAIC with PCNQ in the first paragraph of the Introduction where we have taken this opportunity to divide examples of ribosomopathies into different categories depending on where the variants are found. It now says, “Ribosomopathies arise from variants in either ribosomal proteins or the proteins responsible for creating the mature ribosome and can affect as many as 1 in every 50,000 live births (De Keersmaecker et al., 2015). For example, ribosomopathies such as Diamond-Blackfan anemia are caused by variants in the ribosomal proteins themselves or alternatively, diseases like Progressive cholestasis of northwestern Quebec (PCNQ) (formerly North American Indian childhood cirrhosis) are ribosomopathies caused by a variant in the SSU processome”.**

5-Throughout the paper, there is mention of the importance of WDR43/Utp17. It is not clear (an argument is not made) for why Utp17 is important. Utp17, like most SSU processome proteins, is highly conserved and is an essential protein (as are about 86% of SSU processome proteins). What makes WDR43/Utp17 more important than the other highly conserved and essential SSU processome proteins?

**Yes, we agree that the background requires more justification for why we chose to focus on WDR75/Utp17. Therein, we have added emphasis that the WDR75 is important due to its interaction with pre-rRNA. The introduction now states, “One member of this SSU processome, U three protein 17 (Utp17) or, more commonly, the WD repeat domain 75 (WDR75) is a small nucleolar RNA associated protein that plays an important role in SSU ribosome biogenesis because of its interaction with other proteins and particularly its interaction with the evolutionary conserved motif (ECM) within the pre-rRNA.”**

6-In many places in the ms, there is mention of the 57 beta sheets of WDR43/Utp17. Do the authors mean 57 beta sheets (seems high) or rather 57 beta strands?

**Again, good catch. Fixed. “57 beta sheets” in the introduction and results has been changed to “57 beta strands”.**

7-The naming convention for the Utp proteins is incorrect: it is U three protein.

**Fixed. It now reads, “U three protein 17 (Utp17), a small nucleolar RNA-associated protein…” and “...WDR75 functions in concert with two additional small nucleolar RNA-associated proteins in the SSU processome; U three protein 3 small nucleolar RNA-associated protein 4 (UTP4) and U three protein 3 small nucleolar RNA-associated protein 18 homolog (UTP18)” in the Introduction. We have also followed the reviewer’s lead in using all caps for reference to the protein and leading capital letter followed by lower case for the gene (also in italics).**

8-In line 76, I do not agree with the statement that the ITSs are hypervariable. They are more variable than the main 18S coding sequence, but I would not call them hypervariable.

**Fixed. It now states, “This evolutionarily conserved region is unusual among regions of the rRNA that are removed in the final, mature rRNA since these regions are typically more variable than the main 18S coding sequence (i.e. the internal transcribed spacer region in plants and fungi).”**

9-All of the images in the ms are of very low resolution and difficult to read.

**We attempted to submit high resolution images for review, however this clearly didn’t work. We have tried again. If they remain low resolution, maybe the handling editor can help forward the original image files so the reviewers can thoroughly evaluate the publication quality images and not the blurry ones that came through in the PDF for review. Our apologies for this and I hope it doesn’t happen again.**

10-In lines 96 to 102, there appears to be confusion (as far as I can tell) about the Budkevich reference. A quick review of that reference suggests that it looks at structural rearrangements during translation, not during ribosome assembly. It does so in rabbit but not in cow (contrary to the text). It appears that the use of this reference is out of place and possibly confusing ribosome assembly and translation. Further, the comparison to ribosome assembly in bacteria is maybe not useful as we know that bacterial ribosome assembly is completely different from the process in eukaryotes (bacteria don't have an SSU processome). Lastly, in lines 100-102, this statement is misleading. First, a characterization of ribosome assembly in humans vs yeast does not qualify as widespread evolutionary conservation as yeast and humans are quite closely related. There has been characterization of ribosome assembly in truly distant eukaryotes such as Euglena, trypanosome, and Giardia where that comment would be true of widespread evolutionary conservation.

**Thank you for correcting our mistake. We mistook the reference to bovine liver as additional evidence for the processome, but now see that cows are just the source of the tRNAs used in the Budkevich et al. 2011 study of rabbit ribosomes. More importantly, as you pointed out, this study is about the conformational changes in the pre-translocation ribosome (before it receives the tRNAs), not about how ribosomes originate and mature via the processome. Therefore, we’ve removed all references to Budkevich et al. 2011 and the reference from the literature cited.**

**Instead, when trying to justify the conserved function of ribosomes and likely the proteins and processes responsible for their origin and maturation, we rely on the widespread conservation between humans and yeast which have been separated for approximately 1 billion years. This paragraph now ends with, “Extensive characterization of ribosome maturation has been conducted in yeast demonstrating widespread evolutionary conservation across Eukarya – at least the ~ 1 billion years that mammals and yeast have been separated (reviewed in Woolford & Baserga 2013).”**

11-Line 106 talks about an aim of the study being to uncover the molecular evolutionary history of WDR75/Utp17. It is unclear to me that that can be achieved by only looking at the sequence is mammals. In order to achieve that goals, the sequence of Utp17 would need to be examined throughout Eukaryotes.

**Thank you for the suggestion, we have narrowed the focus of the aim of the study to mammals. The sentence now reads, “This study aims to uncover not only the molecular evolutionary history of this crucial protein in mammals…”**

12-Line 121-while I agree that alternative splicing is important and that it has overall been understudied in ribosome assembly, it is unclear how the study of alternative splicing in WDR75/Utp17 is relevant to ribosomopathies considering that there are no known (as far as I know) ribosomopathies in WDR75/Utp17.

**We removed "relevance to ribosomopathies" and changed it to " protein function...." since we are more interested in the effect of alternative splicing on structure of WDR75. The text now states “...underscoring the complexity of alternative splicing and its relevance to protein function (protein-protein, protein-RNA interactions) in the case of WDR75 (Hsiao et al., 2010). Some diseases are notably caused by alternative splicing. For example…”**

13-In the materials and methods, it is unclear if the multiple sequence alignments/trees where built from DNA or protein sequences. The implication is that they were built from DNA sequences. If so, why DNA and why not protein sequences? Also, where the alignments manually edited (they should be) after having been generated?

**Relevant question, the MSA was built from DNA sequences for multiple reasons. 1. It is possible to translate DNA/RNA to amino acids but not for the reverse. 2. All of the test for selection that we used in our study is based on the codons and estimates of dN/dS are not possible with amino acids alone. 3. Even though are alignment was based on the nucleotides, we frequently translated it to optimize the alignment and infer variable sites.**

**Regarding manual editing of the alignments, we prefer to optimize the alignment parameters and generate the alignment algorithmically, instead of manually editing the alignment. The latter reduces reproducibility of the alignment by others. We have clarified in the methods text, “These parameters were generated through an iterative process of testing multiple parameter settings in order to maximize pairwise identity while minimizing gap insertions…”**

14-In the results section of page 12, why not give the results of the protein alignment? Wouldn't that be more informative that the results of the nucleotide alignment? Also, the protein alignment should be included as a supplemental figure.

**Thank you for the suggestion. We have added the number of amino acids (829) in the average sequence length to the results section.**

**As for showing the entire protein alignment, it is provided along with the nucleotide alignments as Supplementary Data (Table S2) and based on this reviewer’s suggestion, we also included the most relevant portions of the amino acid alignment in the Supplementary Data (Fig. S5) to see the amino acid sequence conservation around the interacting sites (i.e. strong purifying selection).**

15-In Figure 5, why not include the bonobo reference sequence and structure in order to do a better comparison between the full-length human and bonobo sequence/structure?

**Done. Bonobo reference sequence and 3D structure were added to Figure 5 and you can see the 3D structures of the reference vs. the transcript variant aligned in Fig. S3.**

16-In the section on molecular evolution/sequence conservation, how do the authors know that these sequences are conserved due to their particular function (protein-protein, protein-RNA interactions) and not simply conserved due to maintaining the overall structure of WDR75/Utp17?

**Good question and a difficult one to tease apart. Using bioinformatics and molecular evolution, we can only detect the *pattern* of selection in the past. Unfortunately, we don’t have a window into the process of molecular evolution on WDR75 to disentangle whether the negative selection and amino acid conservation is constrained primarily by single protein folding & function versus interactions with other proteins and the rRNA. We have qualified our conclusions as such.**

**Interestingly, Brussa Reis et al. (2019) examined a different subunit that does not interact with the ECM (UTP 6) with evidence for diversifying (positive) selection across 12 mammalian sequences.**

**Therefore, in light of the reviewer’s helpful comment and the Brussa Reis (2019) finding, we have revised the our Discussion statement to read, “While maintaining overall protein structure contributes to sequence conservation of many proteins including WDR75, its interaction with the rRNA’s ECM in the developing SSU is likely an important factor in sequence conservation. A previous study on mammalian UTP6 (one of the many proteins in the SSU processome that does not interact with the ECM) reports evidence of diversifying (positive) selection at 15 amino acid sites across 12 mammalian sequences (Brussa Reis et al., 2019 & their Table S5). The conservation of the WDR75 interacting sites suggests a footprint of purifying selection maintaining the integrity and efficiency of the ribosome assembly process- particularly the binding of pre-RNA in the SSU processome (Singh et al., 2021).”**

17-Table 2 might be more useful if it included the function of the particular amino acids, such as interacting with another protein (in which case, which one?) or RNA?

**Great suggestion. Recommended details added to Table 2.**

18-A protein alignment of WDR75/Utp17 would be helpful. the region identified by the authors (aa 237 to 322), how conserved is it relative to the rest of the protein? An alignment would make this clear.

**We agree that readers should be able to see the relevant portions of the protein alignment. We have added Fig. S5 which shows the amino acid alignment for the sequences studied with disagreements to the human refseq highlighted to emphasize how these sites are mostly conserved compared to the neighboring sites. Here’s the supplemental figure caption:**

**Fig. S5. Mammalian WDR75 amino acid multiple sequence alignment showing high residue conservation at interacting sites. *Homo sapiens* RefSeq (NM_032168) is the reference in yellow at the top. Dots indicate identical amino acids to the reference. Box A shows amino acid sites 237-246 (bp 709-728), B shows amino acid site 288 (bp 862-864), and C contains amino acids 321 and 322 (bp 961-966) (see Fig. 2 for alignment wide location of A,B,C). Note: there is a break in the alignment between regions A and B+C.**

19-Lines 367-370 is partially incorrect. Depletion of most SSU processome proteins in yeast gives a pre-rRNA processing defect (as in, pre-rRNA transcription is normal (production of the 35S) but there is a block in the pre-rRNA processing/cleavage. However, for the members of the UTP-A/t-Utp subcomplex, their depletion results in both a decrease in pre-rRNA transcription (loss of the 35S) AND pre-rRNA processing.

**Fixed. The line now says, “Although depletion of most SSU processome proteins in yeast results in a pre-rRNA processing defect (i.e. they still produce the 35S), however for the members of the UTP-A/t-Utp subcomplex (including UTP17/WDR75), their depletion causes an additional reduction in pre-rRNA transcription (i.e. diminished production of the 35S) as well as reduced pre-rRNA processing. Our results align with the essential nature of WDR75 in not only the processing, but also maintaining the transcription of rRNA during ribosome biogenesis (Freed & Baserga, 2010).”**

20-Line 373-374: are there other alternatively spliced isoforms of WDR75/Utp17? or only the one mentioned with the downstream start codon?

**Many mammalian species included in this analysis have alternative splice isoforms (aka transcript variants). Most are consistent with the human and bonobo showing loss of residues on the N-terminal side of the protein. Therefore, we focused on the human and bonobo as examples (see updated Fig. 5 and new Fig. S3 for more on the human & bonobo 3D structure comparisons).**

21-Lines 383-384 talks about WDR75/Utp17 transcript variants being low expression. What is this based on?

**Our prediction is that the transcript variants will have low expression since the loss of those N-terminal residues changes the secondary and tertiary structure and will likely have deleterious effects on its function. We have clarified this by stating that the prediction remains to be tested.**

**The lines now say, “We predict that in the case of WDR75, the investigated transcript variants will have considerably lower expression compared to their respective reference sequences because of the effects the transcript variant has on secondary and predicted 3D structure (Fig. 5 and Fig. S3), however this remains to be tested.”**

22-The bottom paragraph of page 20 talks about faster substitution rates. Are these faster substitution rates know for those particular organisms, beyond the comment of their short reproductive life cycle? This makes it difficult to determine if WDR75/Utp17 has a high substitution rate in those organisms or if that is a feature of all genes in those organisms.

**Thank you for pointing out another important distinction. While we initially thought the high substitution rates in those organisms was unique to WDR75, we found that removing insertions unique to *Myotis* had no effect on the branch length (substitutions/site). We found that the high substitution rates for *Myomorpha* and *Myotis* is consistent with current literature on mammalian molecular evolution that shorter generation times are correlated with higher substitution rates. To clarify this, we have added the following to the discussion, “The elevated substitution rates observed in *Myotis* and Myomorpha are most likely attributed to their shorter generation times, which are correlated with higher genomic substitution rates across their genome, including WDR75/Utp17.”**

23-Line 418 and elsewhere talks about there being only a single chain of WDR75 in the human SSU processome cryoEM structure. This is likely because WDR75/Utp17 is a single-copy protein within the SSU processome. It is unclear why this statement is made here. Why is this being said?

**WDR75 exists as a single chain within the SSU processome cryoEM structure, because of this, many buried residues are not modelled. We tried to address this by predicting the WDR75 structure in AlphaFold, however the resulting protein had low confidence.**

**We revised the Limitations for clarity, “3D structure analysis was limited to existing PDB files of SSU processomes in which WDR75 is a single chain (out of 71 in humans), existing files were not able to model many of the residues buried in the SSU processome structure including several alpha helices and coils. When predicting the 3D structure of WDR75 using AlphaFold, the alpha helices and coils between helices missing from the PDB were modelled with low (70> pLDDT> 50) to very low confidence (pLDDT< 50). Because of the low confidence, further studies on WDR75 should consider experimental methods to determine the remainder of the monomeric 3D structure.”**

Reviewer #2 Comments:

Reviewer #2: The manuscript provides a comprehensive analysis of the conservation and molecular evolution of the WDR75 protein. The authors conducted a multiple sequence alignment across mammalian species, inferred a phylogenetic tree, and integrated this with both experimentally determined and predicted 3D structures. They report evidence of purifying selection and identify an unusually high substitution rate in bat lineages, attributing this to short generation times. Overall, the manuscript is well written, the approach is sound, and the results could be of interest to others studying this protein.

Major comments

The analysis depends heavily on the Geneious software, which is a paid, proprietary platform. This poses potential limitations for reproducibility, as not all readers may have access to this software. Could the authors elaborate on their choice of Geneious over open-source tools? Was there any specific functionality or feature in Geneious that could not be replicated using freely available software?

**Great point and we are big fans of accessibility which proprietary, pay-to-play software like Geneious seems to contradict. However, when teaching Bioinformatics, the open-source implementations (usually command line) can be a barrier to students’ ability to understand and do bioinformatics. Therefore, I have chosen to use Geneious in my upper division Bioinformatics course where all students get 12 weeks (quarter system) of free access – and frequent extensions and additional licenses are provided upon request of the Geneious support team. This choice, however painful, has greatly improved the quality of work that undergraduates can produce in < 10 weeks (as evidenced by this manuscript?).**

**In terms of reproducibility of the study, most of the Geneious-based methods described in this manuscript are freely available as stand-alone programs. For example, there are freely available alignment algorithms (CLUSTAL, MUSCLE, MAFFT) and tree building software (RAxML, PHYLIP, MEGA) – in addition to the numerous R-based tools in phytools (Revell, 2011 in *Methods in Ecology & Evolution)*. In response to the reviewer’s concern, we have offered all the alignment files in Table S2 which readers are welcome to download and input into their platform of choice. Note: many of our analyses are not Geneious dependent (selection analyses use Datamonkey, Predict Protein for secondary structure, and Alpha Fold are all accessed via a web-servers while PyMOL is freely available for download). We don’t feel that our use of Geneious as a front-end for some analyses presented herein reduces the reproducibility of our study, but appreciate the importance of open-source software and will take that into consideration in future studies.**

Minor comments

Given that multiple sequence alignment is central to the analysis, I recommend making the complete MSA available in Stockholm format in the supplementary materials. This would allow others to scrutinize the alignments more easily and potentially reuse them for future studies.

**Thank you for the suggestion. We have provided all multiple sequence alignments used in this study as FASTA files in the Supplementary Data section since this is the most widespread and universally used format for DNA and amino acid sequence alignments (see Table S2).**

Similarly, since the manuscript involves predicted structures, I suggest including the predicted 3D models in widely accessible formats, such as PDB or mmCIF, as supplementary files. This would be beneficial for reproducibility, especially given that prediction algorithms (such as those used by AlphaFold) may improve or change over time, potentially affecting the reproducibility of the structural analysis.

**Important suggestion. We have included the PDB file names of all the predicted 3D structures included in the study in the text. They can be easily accessed from alpha-fold so no need to provide the entire file.**

Figures 2, 3, 5, 6, and 7 were unreadable in the provided PDF proof. Please ensure that all figures are presented with high resolution and are properly embedded in the final submission.

**Our apologies on behalf of the PLOS ONE robots that downscaled our figure images. We will attempt to ensure the resolution is higher in the revised PDF (they are 300dpi TIFF equivalent when submitted). Note: it is always acceptable to ask the handling editor for higher resolution copies (if you can’t find them yourself in the reviewer portal).**

Typos and Style Corrections

1. In the sentence “While my study offers…”, the wording should be revised to “While this study offers…”.

**Done.**

2. In the sentence “maturation in ‘mammals’ and even ‘eukaryotes’ in comparison to bacteria,” the use of quotation marks around mammals and eukaryotes is unnecessary and may create confusion.

**Done.**

Thank you,

Justen Whittall and Lauren Lee

[jwhittall@scu.edu](mailto:jwhittall@scu.edu)

(831) 332 3389
