# Supplementary material for: WDR75: An essential protein for ribosome assembly undergoing purifying selection
Source: PLoS One. 2025 Feb 11;20(2):e0318395. doi: 10.1371/journal.pone.0318395 (PMC11813130; doi:10.1371/journal.pone.0318395)
Supplement: Attachment — Submitted filename: Response to Reviewers letter.R4 copy.docx [file pone.0318395.s012.docx]

January 13, 2025

Dear PLOS ONE Editor,

Thank you for sharing those suggestions on our manuscript (PONE-D-24-35385) entitled, “WDR75: An essential protein for ribosome assembly undergoing purifying selection.” We have integrated all of your suggestions into this revised version. Below you will find your suggestions followed by our responses in **bold**.

Editor Suggestions

Linked to answer #1 and #2:
Line51. It seems there is a mistake. It is written "(*PCNQ) (formerly North American Indian childhood cirrhosis) are ribosomopathies caused by* ***a variant in the ribosome assembly complex known as the SSU processome****.*" Since the ribosome assembly complex known as the SSU processome is not a gene, I think the sentence should be something similar to "a variant/s in (identify the factor) some factors (if several) participating in the assembly of the first pre-ribosomal particle , or SSU processome"

**Thank you for noticing this misstatement on our part that could lead to some ambiguous interpretation. We have taken your suggestion and updated the sentence to read, “…(PCNQ) (formerly North American Indian childhood cirrhosis) are ribosomopathies associated with a variant in the UTP4 gene, a component of the SSU processome involved in small ribosomal subunit assembly.**

By the way, line 52 contains two dots "processome. ."

**Fixed.**

Linked to answer #3:
Line 65 "*UTP4 and UTP18*",  think about including UTP6 and UTP10 as the tUTP complex seems to associate as a protein complex, composed of 7 proteins in yeast, 5 in mammals (look at our Plos one paper: Pöll, G. et al. In vitro reconstitution of yeast tUTP/UTP A and UTP B subcomplexes provides new insights into their modular architecture. PloS one 9, e114898, 2014). Do not consider the option if you think I am wrong.

**Thank you for continuing to educate us on the tUTP complex and pointing out that UTP6 and UTP10 interact with UTP4 and UTP18 (yet uncertain if it interacts directly with WDR75 in primates though). These multiple, essential interactions likely put additional evolutionary pressure on the molecules constituting the tUTP complex further contributing to the strong purifying selection we detected. Value added. We included your reference in the Introduction. It now reads, “Furthermore, UTP4 and UTP18 associate with UTP6 and UTP10 to support assembly and stabilization of the tUTP complex whose interactions are important for ensuring the overall structure and function of the processome [9].” We renumbered all subsequent references.**

Linked to answer #4:
Line 93 "*maintains essential subunits of RNA polymerase I*" I would suggest "participates in RNA polymerase I transcription" Otherwise it will be missunderstand by a direct influence on the stability of RNA polymerase I subunits, whish is not the case.

**Repaired as suggested.**

Thank you,

Justen Whittall and Lauren Lee

[jwhittall@scu.edu](mailto:jwhittall@scu.edu)

(831) 332 3389
